# Keratin 6A (KRT6A) promotes radioresistance, invasion, and metastasis in lung cancer via p53 signaling pathway

**DOI:** 10.18632/aging.205742

**Published:** 2024-04-17

**Authors:** Qiang Xu, Ziyang Yu, Qiteng Mei, Kejun Shi, Jiaofeng Shen, Guangyu Gao, Songtao Liu, Ming Li

**Affiliations:** 1 Department of Thoracic Surgery, Suzhou Xiangcheng People’s Hospital, Suzhou, Jiangsu, China; 2 Department of Gynaecology and Obstetrics, The Second Affiliated Hospital of Soochow University, Suzhou 215004, China; 3 Department of Oncology, The Second Affiliated Hospital of Soochow University, Suzhou 215004, China; 4 Department of Ultrasound, The Second Affiliated Hospital of Soochow University, Suzhou 215004, China; 5 Department of Pathology, Suzhou Municipal Hospital affiliated to Nanjing Medical University, Suzhou 215008, People’s Republic of China

**Keywords:** KRT6A, lung cancer, GEO, CCK-8, radioresistance

## Abstract

Background: It is reported that the incidence rate and mortality of lung cancer are very high. Therefore, early diagnosis and identification of specific biomarkers are crucial for the clinical treatment of lung cancer. This study aims to comprehensively investigate the prognostic significance of KRT6A in human lung cancer.

Methods: The GEO2R online tool was utilized to analyze the differential expression of mRNA between lung carcinoma tissues and radioresistant tissues in the GSE73095 and GSE197236 datasets. DAVID database was used to perform GO and KEGG enrichment analyses on target genes. The Kaplan-Meier plotter tool was used to analyze the impact of key messenger ribonucleic acid on the survival status of lung cancer. In addition, quantitative real-time polymerase chain reaction (qPCR) was used to investigate the impact of key genes on the phenotype of lung cancer cells. After the knockout, we conducted cell migration and CCK-8 experiments to detect their effects on cell proliferation and invasion.

Results: 40 differentially expressed genes (DEGs) were chosen from GSE73095 and 118 DEGs were chosen from GSE197236. Kaplan-Meier map analysis showed that the overall cancer survival rate of the high-expression KRT6A group was higher than that of the low-expression group (*P* < 0.05). Besides, cell experiments have shown that when the KRT6A gene is downregulated, the proliferation and invasion ability of lung cancer cells is weakened.

Conclusions: Our research concluded that KRT6A may take part in the radioresistance and progression of lung cancer and can be a potential biomarker for lung cancer patients.

## INTRODUCTION

According to statistics, there were approximately 2.2 million new cases and 1.8 million deaths of lung cancer worldwide in 2020. According to histological types, lung cancer can be divided into small cell lung cancer (approximately 15%) and non-small cell lung cancer (NSCLC, approximately 85%) [1]. Non-small cell lung cancer can be further divided into four subtypes: Adenocarcinoma, Squamous cell carcinoma, Large cell carcinoma, and Carcinoid. Among them, Adenocarcinoma of the lung is the most common, accounting for 40% of the total number of lung cancers [2]. The traditional treatment methods for Adenocarcinoma of the lung include surgery, chemotherapy, and radiotherapy [3]. Because Adenocarcinoma of the lung is not easy to find in the early stage, most patients cannot benefit from surgical treatment when diagnosed, and radiotherapy has become the main treatment method for Adenocarcinoma of the lung. However, radiation resistance greatly reduces the radiotherapy effect on patients and affects their radiotherapy prognosis [4, 5]. Therefore, it is of great significance for the clinical treatment of Adenocarcinoma of the lung to explore the characteristics of patients with better radiotherapy prognosis and explore ways to improve radiotherapy sensitivity.

Tumor immunology and immunotherapy provide a new perspective for cancer treatment and are another effective tumor treatment method following traditional therapies [6]. In clinical practice, immune checkpoint inhibitors (ICI) have made breakthroughs in the treatment of lung cancer. Among them, immune checkpoint inhibitors targeting Programmed cell death receptor 1 (PD-1) and its ligand (PD-L1) have been approved for marketing and have achieved satisfactory clinical treatment results [7]. According to reports, TMB is relevant to immune infiltration and the prognosis of various cancers [8]. Previous studies have shown that TMB has become an effective biomarker for many cancer types to predict the efficacy of ICB [9, 10]. However, the overall response rate of ICI is relatively low, and only some patients with Adenocarcinoma of the lung can benefit from ICI treatment [11]. Therefore, it is crucial to identify more immunotherapy targets and elucidate the molecular mechanisms underlying complex immunotherapy responses.

This study is based on the TCGA database and the GEO database to screen for genes that affect lung cancer and radiation sensitivity. It hoped to search for radiation-sensitive radiotherapy targets at the gene level, to provide theoretical support for personalized radiotherapy for lung cancer.

## METHODS

### Database screening

The chip data were obtained from the GEO database of the National Center for Biotechnology Information (NCBI) in the United States (https://www.ncbi.nlm.nih.gov/geo/) and were screened into the GSE73095 and GSE197236 datasets for further analysis. This dataset contains 12 samples, including 6 lung carcinoma tissues and 6 radioresistant tissues. The chip platform is GPL9040 (febit Homo Sapiens miRBase 13.0), and the species is Homo sapiens.

### Difference analysis

We divided the raw data into the normal group and the tumor group and used the GEO2R online tool to perform gene differential expression analysis on the raw data. The screening criteria for differentially expressed genes were adjusted for *P* < 0.01, with | log2FC |>2 as the screening criteria for upper- and lower-regulated genes. Use ggplot2 to draw volcano and heatmaps of differentially expressed genes.

### GO and KEGG pathway analysis

We extracted the differentially expressed genes screened above and used the DAVID online database for Gene Ontology (GO) functional enrichment analysis with the background of Homo sapiens, including biological processes (BP), cellular components (CC), and molecular functions (MF); KEGG (Kyoto encyclopedia of genes and genomes) signaling pathway enrichment analysis was used to screen for relevant biological pathways. After adjustment, *P* < 0.05 was used as a threshold to screen for the main enriched functions and pathways of differentially expressed genes.

### The relationship between DEG expression and clinical characteristics in TCGA

Survival analysis is a method of studying the relationship between influencing factors, survival time, and outcomes. The logarithmic rank test, sometimes also known as the Mantel-Cox test, is based on the assumption that the null hypothesis holds (there is no difference in the survival curves between the two populations), and the theoretical number of deaths calculated based on the number of initial observations and the theoretical probability of death for different survival periods should not differ significantly from the actual number of deaths. Otherwise, the null hypothesis does not hold, and the difference between the two survival curves is considered statistically significant. Generally, *P* < 0.05 has statistical significance, indicating a correlation between different grouping conditions and survival. On the contrary, for survival curves that were initially stretched wide and gradually approached later, the Breslow method is more likely to obtain statistical differences than the log-rank method.

### Immunohistochemical staining

The expression of KRT6A in Adenocarcinoma of the lung tissue was detected by immunohistochemical staining. KRT6A monoclonal antibody was purchased from Boorsen Company (bs-20482R) with a dilution concentration of 1:400, and the operation steps were carried out according to the product manual. (1) Wash the slices in xylene I, xylene II, xylene III, anhydrous ethanol I, anhydrous ethanol II, 85% alcohol, 75% alcohol, and distilled water for 5 minutes each. (2) Place the tissue slices into a repair box and place them in a microwave oven. Bring to a boil on medium heat for 10 minutes, then cease-fire for 5 minutes. Keep warm and then switch to medium-low heat for 5 minutes, then cease-fire for 2 minutes, and then switch to medium-low heat for 5 minutes. Do not dry the slices. Wash PBS 3 times for 5 minutes each time. (3) Incubate in a 3% hydrogen peroxide solution for 25 minutes and wash with PBS three times for 5 minutes each time. (4) Cover with 3% BSA dropwise and seal at room temperature for 30 minutes. (5) Add an antibody dropwise and lay the slices flat in a wet box at 4°C for overnight incubation. (6) Shake and wash on the shaker three times, each time for 5 minutes. Add a secondary antibody to cover the tissue and incubate at room temperature for 50 minutes. (7) Wash PBS 3 times for 5 minutes each time. Add DAB color solution dropwise, control the color development time under a microscope, and rinse the slices with tap water to terminate the color development. (8) Resustaining with hematoxylin for about 3 minutes, washing with tap water, differentiation of hematoxylin differentiation solution for a few seconds, rinsing with tap water, hematoxylin blue returning solution, and rinsing with running water. (9) Dehydration and sealing: Place the slices in 75% alcohol, 85% alcohol, anhydrous ethanol I, anhydrous ethanol II, n-butanol, and xylene I for dehydration and transparency (each for 5 minutes), and dry them slightly before sealing with neutral gum. (10) Result determination: Referring to previous determination methods, randomly select 5 high-power microscopes for each slice (×400) field of view, counting 200 cells per field, and positive cells with brownish-yellow particles in the cytoplasm. 0 points: No positive cells; 1 point: Positive cells <25%; 2 points: Positive cells 26–50%; 3 points: positive cells 51–75%; 4 points: Positive cells >75%.

### Cell culture and transfection

A549 cells (belonging to adherent cells) culture conditions: First, prepare a complete culture medium containing 89% DMEM high glucose medium+10% FBS (fetal bovine serum)+1% dual antibodies (penicillin and streptomycin), then prepare A549 cell suspension, transfer it to a cell culture bottle, and finally place it in a cell culture incubator. The incubator conditions are set at 37°C, 5% carbon dioxide (CO_2_), and saturated humidity. Carefully observe the cultured cells under a microscope every day and analyze their growth status. Choose to replace the complete culture medium every 2–3 days. When the fusion degree of cell growth is observed to account for about 80%, cell passage culture can be carried out.

### Cell viability and invasion assays

We divided the experimental groups into the KRT6A-A549 group and Ctrl-A549 group. In addition, KRT6A-A549 group and Ctrl-A549 group cells were prepared into cell suspensions, counted, and adjusted to a concentration of 5 × 10^5^ pieces/mL. KRT6A-A549 group and Ctrl-A549 group each have 3 Transwell chambers, which are grouped and placed in a new 24-well plate. After 1 day of cultivation, we removed the 24 well plate, clamped the Transwell chamber with tweezers, discarded the culture medium in the well, washed with PBS, fixed with 4% paraformaldehyde for 30 minutes, slowly washed with PBS 2–3 times, then added an appropriate amount of crystal violet staining solution, stained for 20 minutes, washed with PBS 2–3 times, and wiped off the upper layer of cells that had not been transferred in the chamber, carefully removed each Transwell chamber membrane using tweezers, moved it to the center of the slide, and sealed the slide with neutral resin. After sealing, we took photos under a microscope for observation and randomly selected a field of view to take 100 photos × take 3 photos and analyzed them after counting.

### Wound healing assay

This article designed experimental groups as the KRT6A-A549 group and the Ctrl-A549 group. We prepared cell suspensions from KRT6A-A549 and Ctrl-A549 groups, counted, and adjusted the concentration to 1 × 10^5^ pieces/mL. After 24 hours of cultivation, we aspirated the complete medium from the 6-well plate and then added a serum-free medium. After scratching, we washed the cells with PBS 2-3 times to remove them. Then, we added serum-free culture medium and placed the 6-well plate in the incubator for constant-temperature cultivation. We selected 0, 12, 24, and 48 hours and took photos under a fluorescence microscope. Based on the photos taken, we opened the images using ImageJ software and calculated the average scratch area of the cells. We analyzed the migration rates of each group of cells and took the average of the three times. Cell migration rate (wound healing rate) = (initial scratch area-scratch area at time t)/initial scratch area.

### Statistical methods

We used SPSS 2.0 statistical software, and the measurement data that did not follow a normal distribution were represented by the median (lower quartile, upper quartile). The Mann-Whitney *U*-test was used for comparison between the two groups, and the Kaplan-Meier survival curve method was used for survival analysis. *P* < 0.05 indicates statistically significant differences.

### Availability of data and materials

The datasets used and/or analyzed during the current study are available from the corresponding author upon reasonable request.

## RESULTS

### Identification of DEGs

**Figure 1:**
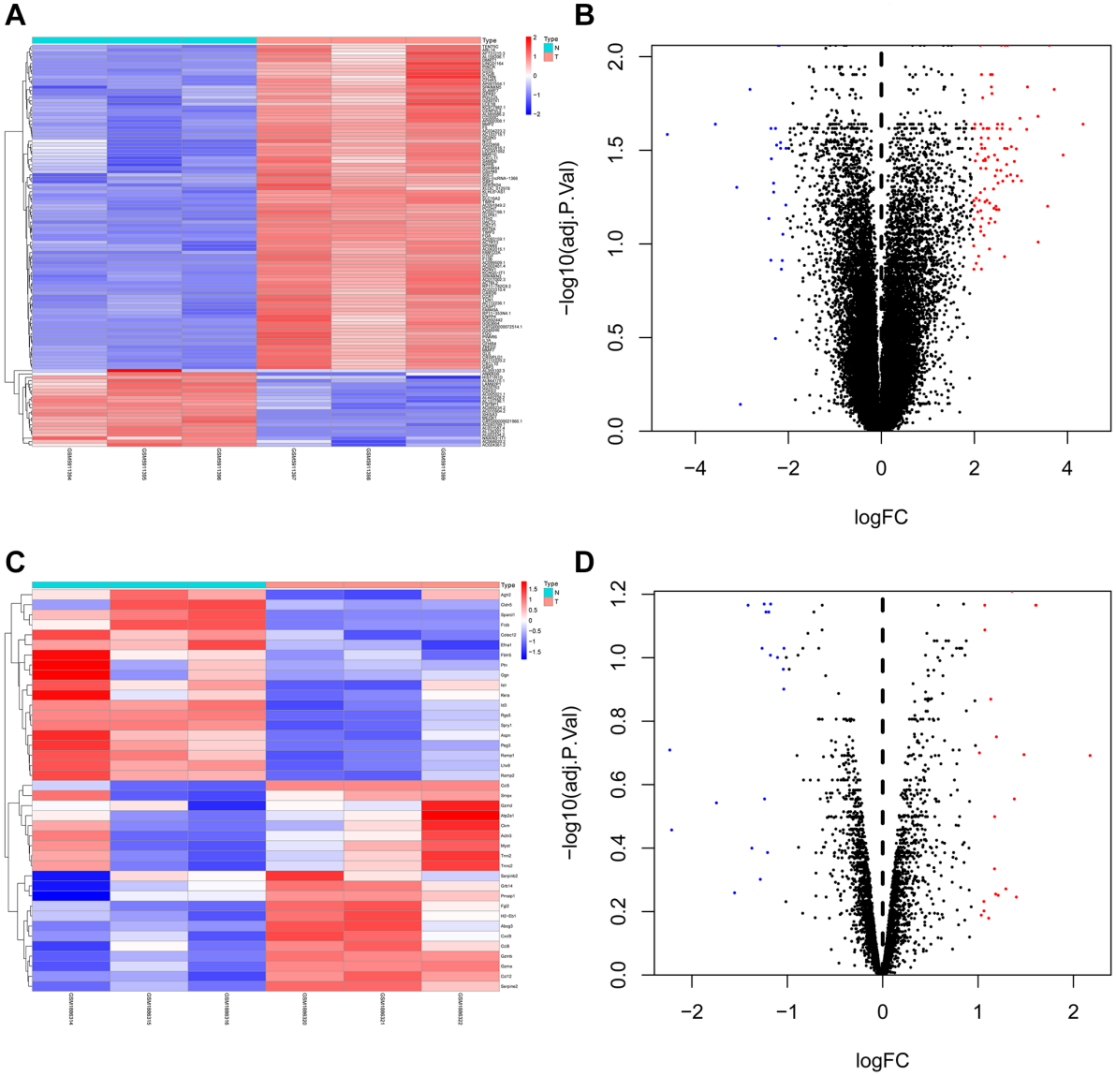
**Heat map and volcano map of differentially expressed genes of GSE73095 and GSE197236.** (**A**, **B**) Heat map and volcano map of DEGs in GSE73095. (**C**, **D**) Heat map and volcano map of DEGs in GSE197236.

**Figure 2:**
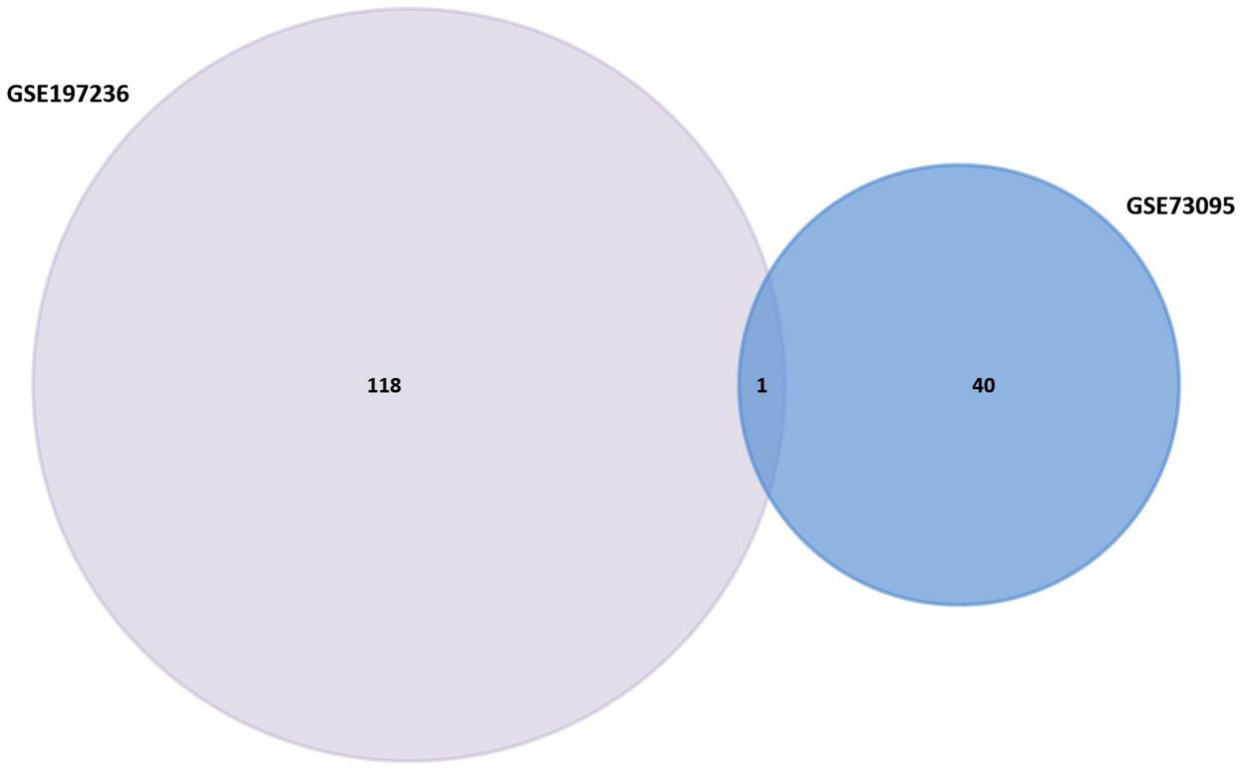
Venn diagram of GSE158662 and GSE194261.

GEO2R was utilized to research the DEGs from GSE73095 and GSE197236. After screening, 40 DEGs such as KRT6A, FGA, SHISA3, C3, TIMP3, GLIPR1, SLC16A2, BMP2, and CRCT1 were obtained from GSE73095. By the same method, 118 DEGs such as GZMB, SPARCL1, RGS5, CCL12, LHX9, CCL5, SERPINE2, SPRY1, ID3, and GZMA were obtained from GSE197236 (Figure 1). Using the Venn plot, we select one common DEG for further research (Figure 2).

### Gene Ontology and KEGG enrichment analyses

**Figure 3:**
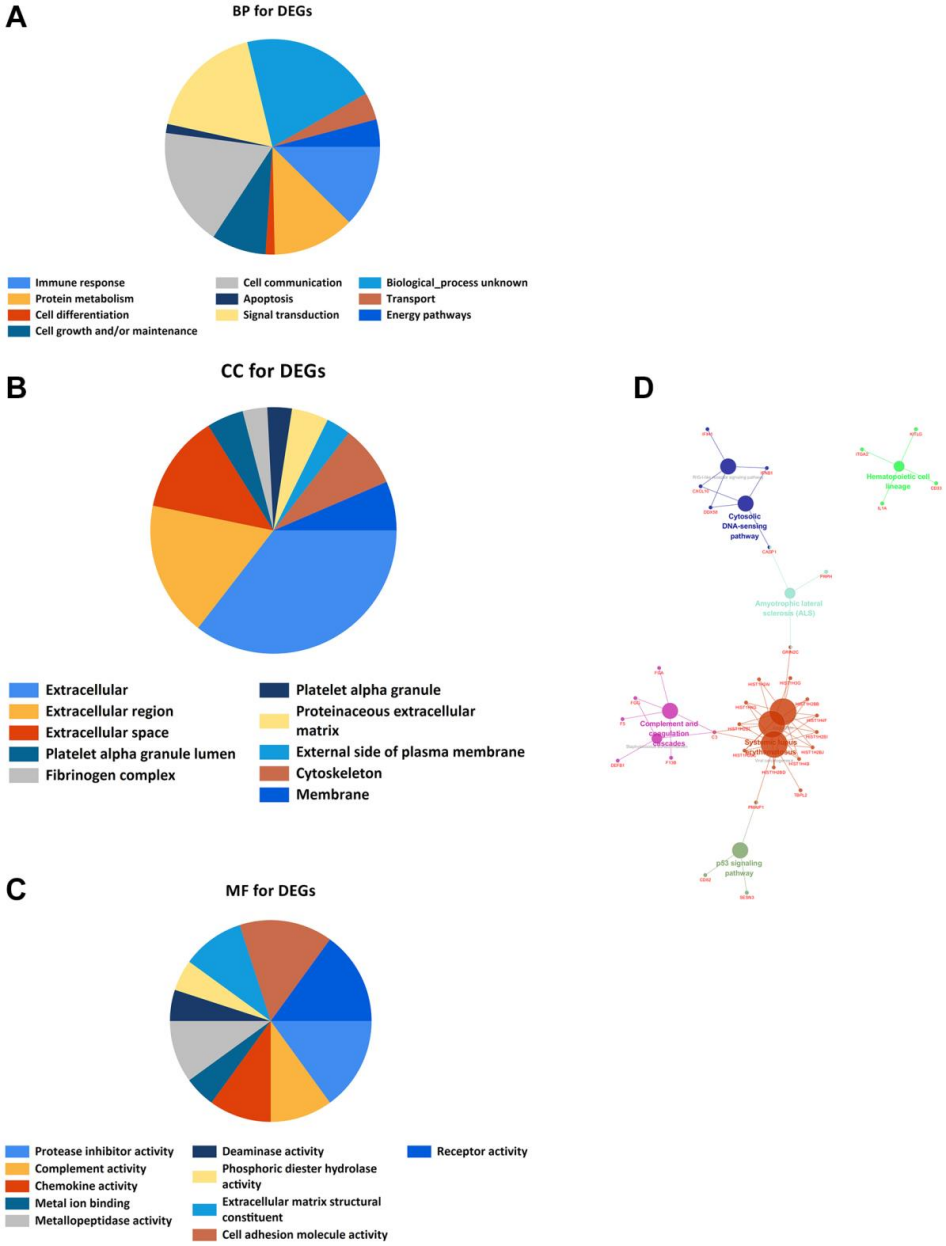
**GO and KEGG enrichment analyses.** (**A**) Bioprocess enrichment analysis; (**B**) Cell component enrichment analysis; (**C**) Molecular functional enrichment analysis; (**D**) KEGG signaling pathway enrichment analysis.

Xiantao Academic Software can conduct common bioinformatics statistical analyses and result in visualization online. GO and KEGG analyses showed that DEGs are mostly enriched in Immune response, Protein metabolism, Cell Differentiation, Cell growth and/or maintenance, Fibrinogen complex, Platelet alpha granule, Platelet alpha granule lumen, Protease inhibitor activity, Complement activity, Chemokine activity, p53 signaling pathway, Hematopoietic cell lineage, Amyotrophic lateral sclerosis (ALS), RIG-I-like receptor signaling pathway, Cytosolic DNA-sensing pathway, Complement, and coagulation cascades, Staphylococcus aureus infection, Alcoholism, Viral carcinogenesis, and Systemic lupus erythematosus (Figure 3).

### The relationship between gene expression and NSCLC overall survival

**Figure 4:**
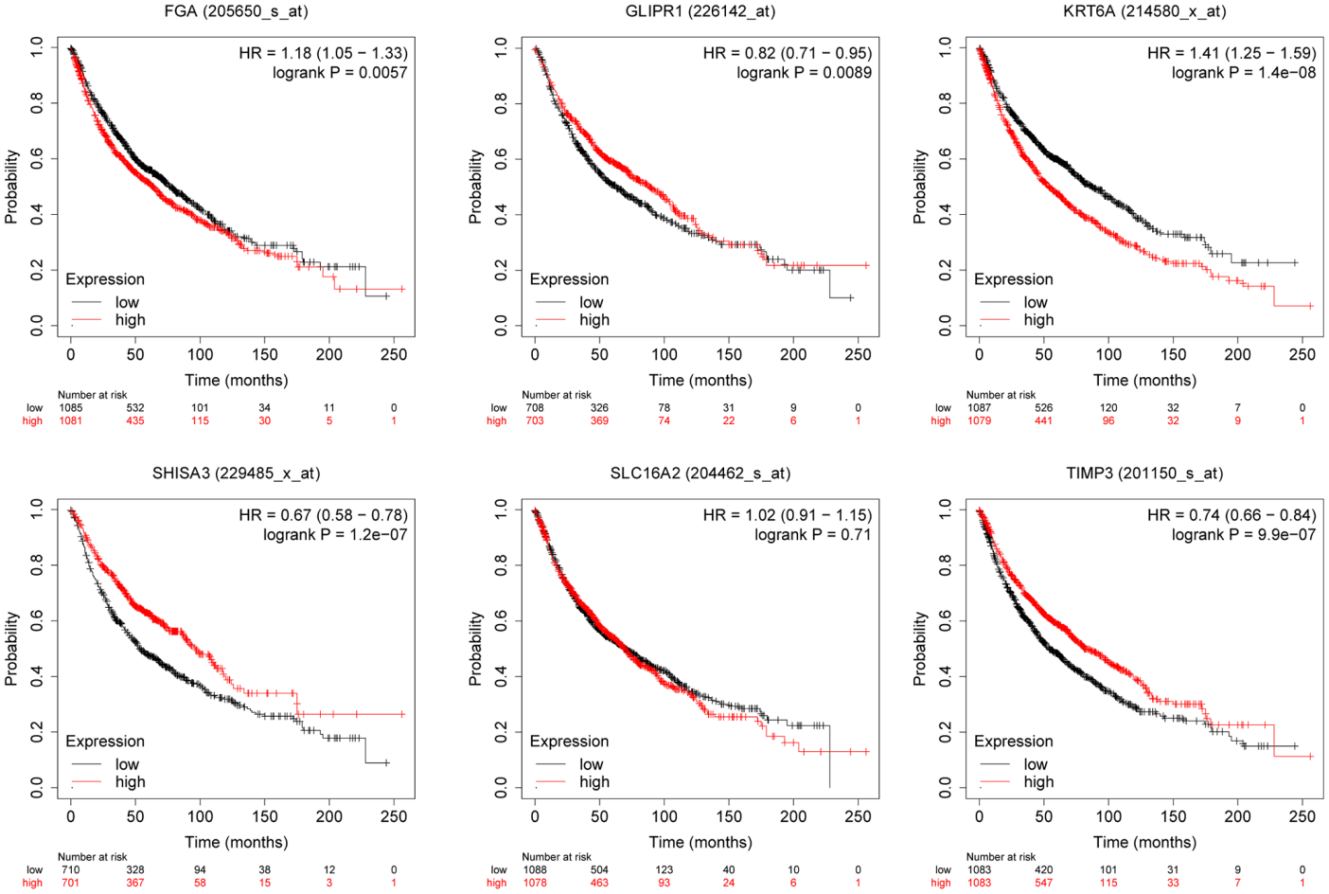
The relationship between the expression level of selected genes and overall survival of lung cancer patients.

We evaluated the relationship between the expression of six differentially expressed genes screened and the prognosis of lung cancer patients through the Kaplan-Meier plotter database. The research indicated that KRT6A was associated with lung cancer OS (Figure 4). Therefore, we selected KRT6A for further research.

### Immunohistochemical study

**Figure 5:**
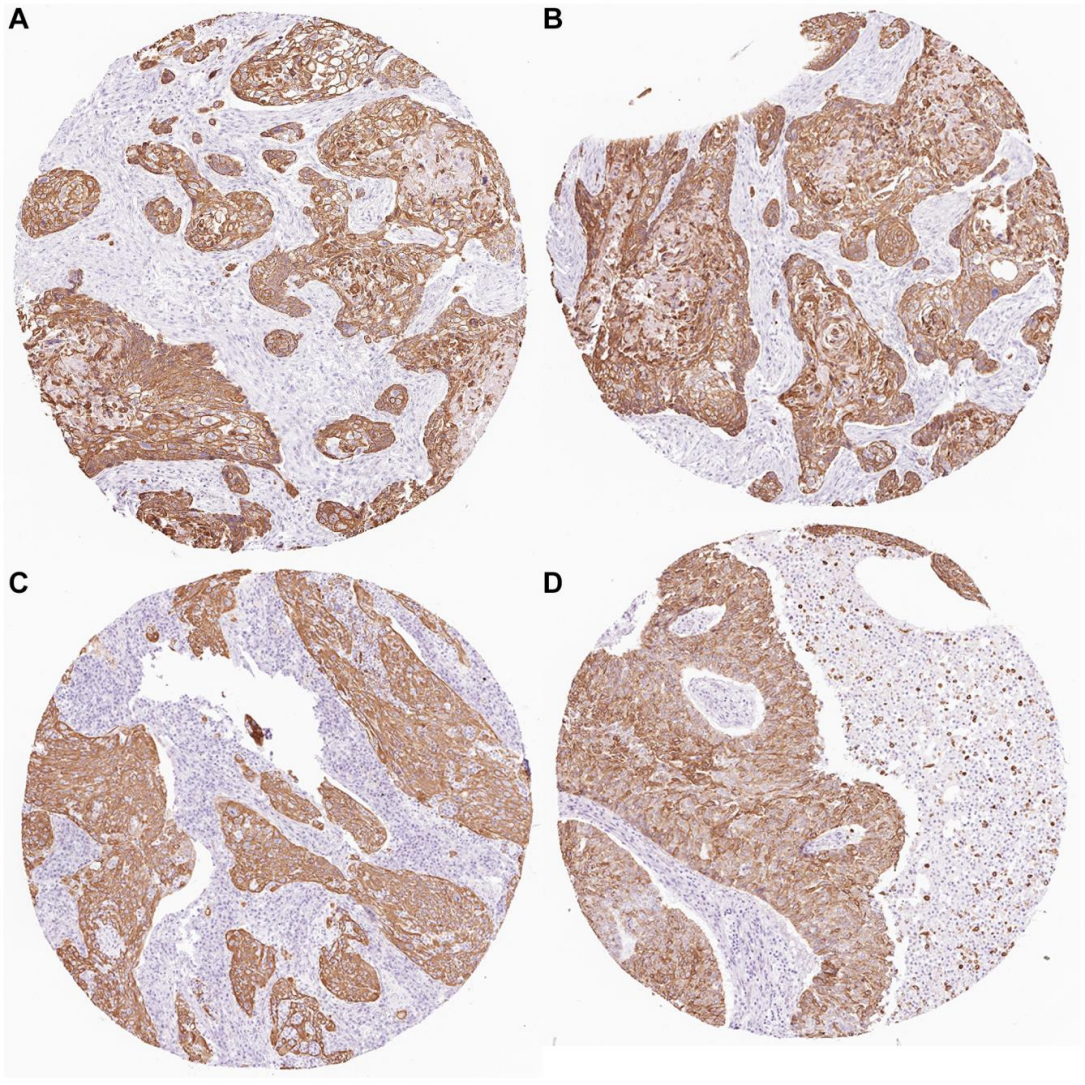
**KRT6A expression in NSCLC tissues.** (**A**–**D**) KRT6A is highly expressed in lung cancer tissues.

The HPA (Human Protein Atlas) database is based on proteomics, transcriptomics, and systems biology data, and can plot tissues, cells, organs, etc. It not only includes protein expression in tumor tissues but also normal tissues, and also views the survival curve of tumor patients. For further evaluation of the expression of KRT6A, the HPA database was used for immunohistochemical detection of gene expression levels. From the perspective of image and statistics, KRT6A is significantly expressed in NSCLC tissue (Figure 5).

### The relationship between clinical characteristics and KRT6A expression of lung cancer

We obtained clinical and gene expression information of non-small cell lung cancer patients from Xiantao software, including TNM staging, age, survival rate, and primary treatment outcomes (Table 1). TNM staging system is currently the most commonly used tumor staging system internationally, and it is also the standard method for staging malignant tumors in clinical practice. T: The scope and size of the primary tumor. N: Lymph node dissemination. M: Is there a transfer. The outcomes concluded that the TNM stage (*P* < 0.05) and Primary therapy outcome (*P* < 0.05) were related to the OS of patients with NSCLC (Table 2).

**Table 1:** Relationship between the expression level of KRT6A and clinical features.

**Characteristic**	**Low expression of KRT6A**	**High expression of KRT6A**	***p***
*n*	187	188	0.277
T stage, *n* (%)			
T1	8 (2.2%)	11 (3%)	0.672
T2	37 (10.1%)	43 (11.7%)	
T3	80 (21.8%)	88 (24%)	
T4	58 (15.8%)	42 (11.4%)	
*N* stage, *n* (%)			
N0	60 (16.8%)	51 (14.3%)	0.382
N1	45 (12.6%)	52 (14.6%)	
N2	36 (10.1%)	39 (10.9%)	
N3	39 (10.9%)	35 (9.8%)	
M stage, *n* (%)			
M0	169 (47.6%)	161 (45.4%)	0.961
M1	10 (2.8%)	15 (4.2%)	
Primary therapy outcome, *n* (%)			
PD	32 (10.1%)	33 (10.4%)	0.421
SD	8 (2.5%)	9 (2.8%)	
PR	2 (0.6%)	2 (0.6%)	
CR	120 (37.9%)	111 (35%)	
OS event, *n* (%)			
Alive	118 (31.5%)	110 (29.3%)	0.452
Dead	69 (18.4%)	78 (20.8%)	
Age, median (IQR)	67 (58, 73)	67.5 (59, 73.75)	

**Table 2:** The association between OS and the expression level of KRT6A was studied by univariate and multivariate Cox regression.

**Characteristics**	**Total (*N*)**	**Univariate analysis**		**Multivariate analysis**	
		**Hazard ratio (95% CI)**	***P*-value**	**Hazard ratio (95% CI)**	***P*-value**
KRT6A	370	1.751 (1.190–1.915)	0.001		
T stage	362				
T1	18	Reference			
T3 and T4 and T2	344	8.829 (1.234–63.151)	0.030	22692221.327 (0.000-Inf)	0.994
N stage	352				
N0	107	Reference			
N1 and N2 and N3	245	1.925 (1.264–2.931)	0.002	1.398 (0.860-2.273)	0.177
M stage	352				
M0	327	Reference			
M1	25	2.254 (1.295–3.924)	0.004	1.691 (0.874-3.274)	0.119
Primary therapy outcome	313				
SD and PR and CR	249	Reference			
PD	64	4.147 (2.843–6.047)	<0.001	3.532 (2.386-5.229)	<0.001

### Knockdown of KRT6A suppresses NSCLC cell growth

**Figure 6:**
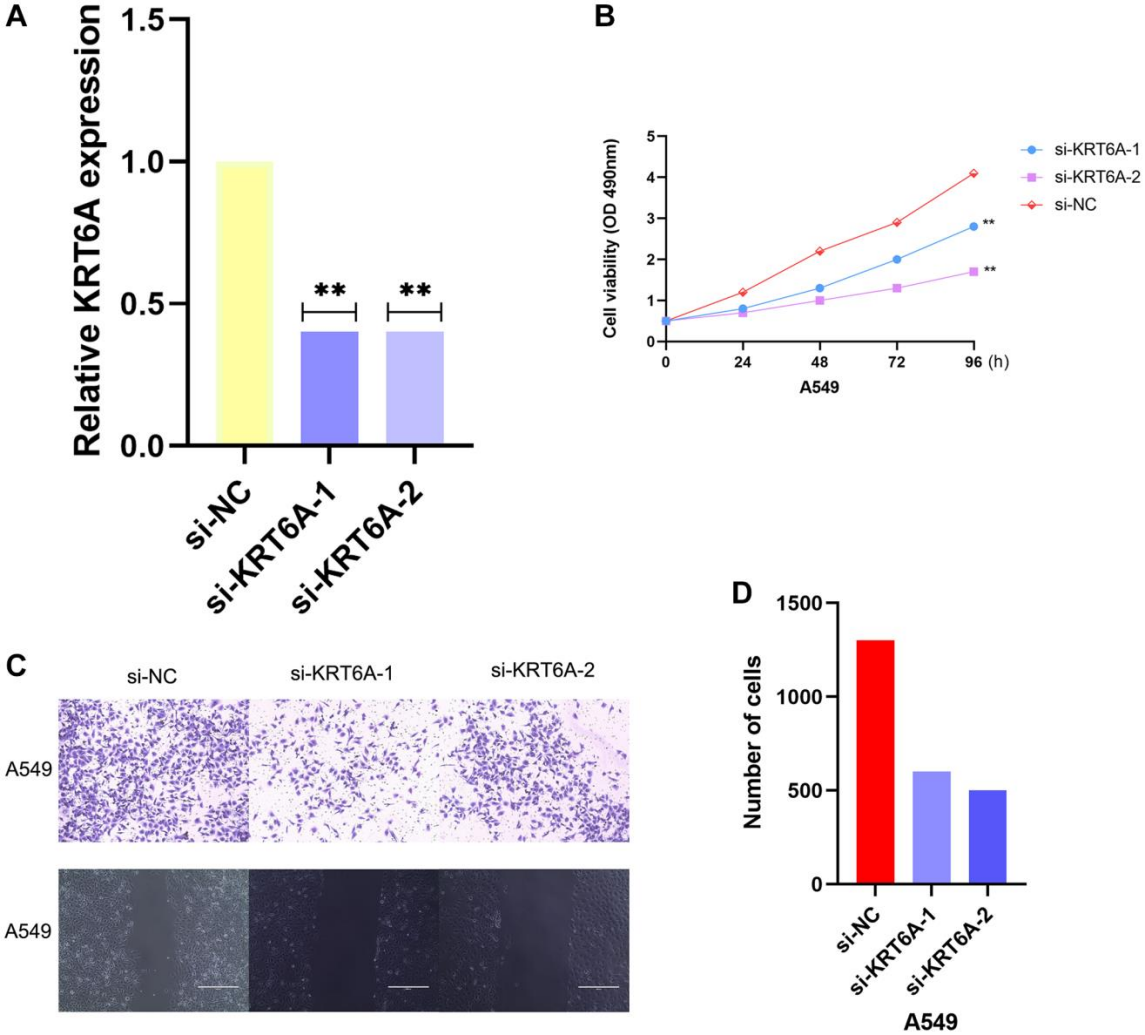
**Effects of KRT6A knockdown on NSCLC cell viability and migratory capacity *in vitro*.** (**A**) KRT6A expression in A549 cell transfected with negative control siRNA (si-NC) or siRNAs targeting KRT6A (si-KRT6A #1 and #2) for 48 h, *n* = 3 for each group. (**B**) Cell viability was assessed using a CCK-8 assay in A549 cell transfected with si-NC or si-KRT6A #1 and #2 for 48 h, *n* = 6 for each group. (**C**, **D**) Transwell invasion assay was performed to determine the invasion ability of si-KRT6A-transfected A549 cell for 48 h, *n* = 3 for each group. ^*^P < 0:05, ^**^P < 0:01 and ^***^P < 0:001 indicate significant differences.

We downregulated the expression of KRT6A by transfecting two independent siRNAs to investigate its potential biological function in NSCLC cells. Both siRNAs obviously reduced the expression of KRT6A (Figure 6A). When cultured for 12, 24, 48, and 72 hours, the OD450 values of the KRT6A-A549 group were 0.727 ± 0.038, 0.820 ± 0.020, 1.293 ± 0.025, and 1.638 ± 0.029, respectively, while those of the Ctrl-A549 group were 0.794 ± 0.014, 0.923 ± 0.024, and 1.793 ± 0.020, respectively, while those of another KRT6A-A549 group were 0.794 ± 0.014, 0.923 ± 0.024, 1.793 ± 0.020, and 2.369 ± 0.026, the blank group was 1.142 ± 0.003, 2.138 ± 0.055, 2.819 ± 0.052, and 4.167 ± 0.027. Compared with the Ctrl-A549 group, the proliferation of KRT6A-A549 group cells was significantly slowed down, and the difference was statistically significant (*P* < 0.05) (Figure 6B). We inoculated the KRT6A-A549 group and Ctrl-A549 group cells into a 6-well plate and cultured them for 14 days. The colony numbers of the Ctrl-A549 group and KRT6A-A549 group cells were (157 ± 14) and (55 ± 9), respectively. The difference in the number of cell colonies between the two groups was statistically significant (*P* < 0.05). The number of migrating cells in the KRT6A-A549 group and Ctrl-A549 group was (521 ± 8) and (533 ± 17), respectively. The migration number of the KRT6A-A549 group was significantly lower than that of the Ctrl-A549 group, and the difference was statistically significant (*P* < 0.05). (Figure 6C, 6D).

## DISCUSSION

Adenocarcinoma of the lung is the most common pathological subtype of lung cancer. Over the years, scholars have found many unique characteristics of genetic changes and prognostic factors. However, due to the unclear pathogenesis, the mortality rate of LUAD patients is still high, and the treatment results are not satisfactory. There are different treatment schemes for Adenocarcinoma of the lung with different stages, and surgery is still the first treatment scheme for early patients, which can significantly improve the prognosis of patients [12, 13]. However, many patients with Adenocarcinoma of the lung are locally advanced or metastasized at the time of diagnosis. At this time, radiotherapy and chemotherapy become the main treatment methods [14]. In recent years, NSCLC, especially Adenocarcinoma of the lung, has made great progress in molecular targeted therapy and immunotherapy, but its survival rate is still very poor, with a 5-year survival rate of less than 18% [15]. Due to the large side effects of chemotherapy, many patients cannot tolerate it. Radiotherapy is more important for patients with Adenocarcinoma of the lung. However, the radiotherapy effect varies for each patient, and the most important reason is the impact of radiation resistance. Therefore, it is particularly urgent to determine accurate biological prognostic markers for radiotherapy to improve the poor prognosis of Adenocarcinoma in lung cancer patients. TMB refers to the number of non-synonymous mutations per megabase pair (Mb) of somatic cells in a specific genomic region, which indirectly reflects the ability and degree of tumors to produce new antigens, and can predict the effect of immunotherapy for a variety of tumors. Tumor gene mutations are considered a prerequisite for anti-tumor immunotherapy. ICIs such as PD-1, PDL-1, and CTLA-4 antibodies can restore anti-tumor immune response by antagonizing T cell activation inhibitory molecules and promoting T cell recognition of tumors [16–18]. As a biomarker of the therapeutic effect of ICI, TMB’s predictive ability is being studied in clinical trials of various tumor types and has been proven to be effective in a variety of cancers, such as lung cancer and melanoma [19]. With the development of data analysis technology, many studies have proposed immune-related gene models that can predict prognosis and provide potential targets for immunotherapy in LUAD patients [20–22], but there are few studies based on TMB to establish prognosis models. In this study, we analyzed the situation of TMB in patients with Adenocarcinoma of the lung, screened out the gene modules most related to TMB through WGCNA analysis, and conducted enrichment analysis to find that most genes in the modules are closely related to cell cycle, DNA repair, replication, etc. In addition, based on univariate regression analysis, LASSO analysis, and multivariate regression analysis, a dual gene model (ANLN and E2F7) with good prognosis prediction ability was constructed. It has been proven that this dual gene model is an independent factor affecting the prognosis of Adenocarcinoma of the lung. By utilizing the complementary value of these two genes and clinical features, we combined these factors to construct a new column chart, providing a more accurate survival prediction method. The radiation damage caused by radiotherapy is closely related to the cell cycle. Ionizing radiation can cause various types of DNA damage: DNA protein cross-linking, DNA single-strand break, Double-strand breaks, base damage, or shedding [23, 24]. After ionizing radiation, multiple pathways (mostly related to the cell cycle) are involved in maintaining genetic integrity [25]. Since the 1960s, scholars have been studying the dependence of radiosensitivity on the pericellular period. The cell cycle is mainly divided into G0/G1, S, and G2/M phases. Current research suggests that the response of tumor cells to ionizing radiation depends on their location in the cell cycle. Cells at different periods have different radiosensitivity, with the G2/M phase having the highest radiosensitivity and the G0/G1 and S phases having poor radiosensitivity [26]. These preliminary findings suggest that blocking tumor cells in the cell cycle with high radiosensitivity would be a potential method to improve the efficacy of radiation therapy. More and more studies have shown that blocking cells during the period of highest radiosensitivity (G2/M) can improve radiosensitivity. Many radiosensitizers, such as Fenofibrate [27], Sunitinib [28], and Salinomycin [29], play a radiosensitization role in different tumors by inducing G2/M phase block. KRT6A is closely related to cell division. KRT6A can regulate cell division by combining with different protein components, including Actin, myosin, cytokinin, etc. In the process of cell division, KRT6A improves the contraction efficiency of Actin and promotes cell proliferation by regulating Actin bundles [30].

Cytolytic proteins are considered important regulatory factors in cell division and mechanical transduction processes, and KRT6A can bind to them to regulate cell proliferation, differentiation, and migration [31]. More and more studies have confirmed a close relationship between KRT6A and tumors, showing high expression in various tumor tissues. The abnormal expression of KRT6A can cause abnormal cell division, leading to the occurrence of tumors. Meanwhile, KRT6A plays an important role in the proliferation, migration, and infiltration of tumor cells. By regulating the expression of KRT6A, the speed of cell division can be controlled, thereby regulating its proliferation ability. Previous studies have shown that KRT6A can promote tumor cell proliferation and metastasis by remodeling Cytoskeleton, promoting epithelial-mesenchymal transition (EMT) and Cell migration. There are also studies suggesting that KRT6A may be the origin of a tyrosine highly correlated with tumor occurrence and development [32, 33].

Although this study has achieved some positive results, there are still some problems. First of all, this risk model is based on a public database, and its predictive ability needs to be further verified in a Randomized controlled trial. Secondly, in the univariate analysis of prognosis, the HR of N0-1 is 5.655 compared with N2-3, which may be contrary to common sense and may be related to the small number of follow-up patients. This result needs to be verified in large sample data. The impact of KRT6A on radiotherapy prognosis may be due to differences in radiation sensitivity caused by different cell cycle distributions, which need to be confirmed in cell experiments and animal experiments.

## CONCLUSIONS

In this article, bioinformatics methods were used to analyze the differentially expressed genes in NSCLC and normal tissues. KRT6A were selected as latent biomarkers of NSCLC. However, we need more cell experiments to prove it.
